# Gαq Is the Specific Mediator of PAR-1 Transactivation of Kinase Receptors in Vascular Smooth Muscle Cells

**DOI:** 10.3390/ijms232214425

**Published:** 2022-11-20

**Authors:** Danielle Kamato, Mai Gabr, Hirushi Kumarapperuma, Zheng J. Chia, Wenhua Zheng, Suowen Xu, Narin Osman, Peter J. Little

**Affiliations:** 1 Discovery Biology, Griffith Institute for Drug Discovery, Griffith University, Nathan, QLD 4111, Australia; 2 School of Environment and Science, Griffith University, Nathan, QLD 4111, Australia; 3 School of Pharmacy, Pharmacy Australia Centre of Excellence, The University of Queensland, Woolloongabba, QLD 4102, Australia; 4 Centre of Reproduction, Development & Aging and Institute of Translation Medicine, Faculty of Health Sciences, University of Macau, Taipa, Macau 999078, China; 5 Institute of Endocrine and Metabolic Diseases, The First Affiliated Hospital of University of Science and Technology of China, Hefei 230052, China; 6 School of Health and Biomedical Sciences, RMIT University, Bundoora, VIC 3083, Australia; 7 Department of Pharmacy, Guangzhou Xinhua University, Guangzhou 510520, China; 8 Sunshine Coast Health Institute, University of the Sunshine Coast, Birtinya, QLD 4575, Australia

**Keywords:** epidermal growth factor receptor, transforming growth factor type I receptor, transactivation dependent, thrombin, GPCR, proteoglycans

## Abstract

Aims: G protein-coupled receptor (GPCR) transactivation of kinase receptors greatly expands the actions attributable to GPCRs. Thrombin, via its cognate GPCR, protease-activated receptor (PAR)-1, transactivates tyrosine and serine/threonine kinase receptors, specifically the epidermal growth factor receptor and transforming growth factor-β receptor, respectively. PAR-1 transactivation-dependent signalling leads to the modification of lipid-binding proteoglycans involved in the retention of lipids and the development of atherosclerosis. The mechanisms of GPCR transactivation of kinase receptors are distinct. We aimed to investigate the role of proximal G proteins in transactivation-dependent signalling. Main Methods: Using pharmacological and molecular approaches, we studied the role of the G⍺ subunits, G⍺q and G⍺11, in the context of PAR-1 transactivation-dependent signalling leading to proteoglycan modifications. Key Findings: Pan G⍺q subunit inhibitor UBO-QIC/FR900359 inhibited PAR-1 transactivation of kinase receptors and proteoglycans modification. The G⍺q/11 inhibitor YM254890 did not affect PAR-1 transactivation pathways. Molecular approaches revealed that of the two highly homogenous G⍺q members, G⍺q and G⍺11, only the G⍺q was involved in regulating PAR-1 mediated proteoglycan modification. Although G⍺q and G⍺11 share approximately 90% homology at the protein level, we show that the two isoforms exhibit different functional roles. Significance: Our findings may be extrapolated to other GPCRs involved in vascular pathology and highlight the need for novel pharmacological tools to assess the role of G proteins in GPCR signalling to expand the preeminent position of GPCRs in human therapeutics.

## 1. Introduction

G protein-coupled receptors (GPCR) constitute the targets of the most prominent class of approved drugs [1]. GPCR signalling is a foundation area of cellular signalling and a long-term focus of pharmacological studies to elucidate diverse physiological pathways and address pathophysiological processes. Classic GPCR signalling, also termed transactivation independent signalling [2], proceeds from agonist activation of the cognate receptor, activation of G protein(s), followed by a plethora of downstream biological processes. GPCRs can also signal via transactivation-dependent mechanisms involving the activation of a second class of cell surface receptors [3,4], which vastly expands the actions attributable to GPCRs. Original observations showed that GPCRs can transactivate tyrosine kinase receptors (TKR) [5]; however, our team identified that GPCRs could also transactivate serine/threonine kinase receptors (S/TKR) via distinct mechanisms [6,7,8]. Our genome studies demonstrate that transactivation-dependent signalling is quantitatively equally important to transactivation-independent signalling [9]. We observed that GPCR transactivation of TKR, epidermal growth factor receptor (EGFR) is equally as crucial to transactivation of S/TKR, transforming growth factor (TGF)-β receptor (TGFBR1) with each receptor pathway accounting for approximately 25% of total genes regulated by thrombin activation of its cognate GPCR, protease-activated receptor (PAR)-1.

There has been an enormous amount of work done to characterise the mechanisms involved in GPCR transactivation-dependent signalling; although a lot is known, there are aspects yet to be defined [10]. GPCR transactivation of TKR, specifically EGFR, consists of the activation of membrane-bound matrix metalloproteinase (MMP), resulting in the cleavage and release of heparin-binding EGF-like growth factor (HB-EGF) that leads to the cognate activation of the EGFR and downstream signalling intermediates (phospho-Erk) leading to gene transcription [5,11]. GPCR transactivation of the S/TKR, specifically TGFBR1, occurs via a rearrangement of the cytoskeleton and activation of ROCK and cell surface integrins, leading to conformational changes in the large latent complex (LLC). The TGF-β ligand is released from the LLC and binds to the TGFBR1 to activate the associated receptor Smads (phospho-Smad) [12]. The pathways of transactivation-dependent pathways are mechanistically distinct, thus rendering it very difficult to identify a single biochemical target that inhibits all transactivation-dependent signalling for therapeutic exploitation.

Atherosclerosis commences with the binding of lipoproteins to extracellular proteoglycans in the vessel wall. Proteoglycans contain a glycosaminoglycan (GAG) chain that, when modified, can bind, and retain passing lipoproteins as one of the key initiating steps in the development of the atherosclerosis [13,14]. In vascular smooth muscle cells (VSMCs), we have found that GPCR agonist thrombin via PAR-1 transactivates both the EGFR and TGFBR1 to stimulate the proteoglycan synthesis [6,15] and the genes involved in the elongation of the lipid-binding GAG chains on proteoglycans [7,8]. Therefore, the common signalling intermediate of GPCR transactivation-dependent signalling, potentially proximal to the GPCR, could identify a therapeutic target to prevent and treat atherosclerosis. One prominent possibility for a common target are G proteins which mediate GPCR signalling, so we have explored the role of G proteins in GPCR transactivation-dependent using pharmacological and molecular tools.

G proteins are categorised into four main classical subfamilies according to their ⍺ subunit G⍺q, G⍺s, G⍺i, G⍺12/13 [16,17,18]. G⍺q is further divided into four classes; among them, Gαq and Gα11 isoforms are the most ubiquitously expressed and will be the focus of this paper. These two isoforms are almost 90% similar in their amino acid sequence, but the possibility of exhibiting different functional roles has not been investigated. Over the past three decades, the commercial availability of two naturally derived G⍺ protein antagonists, UBO-QIC (FR300259) [19] and YM254890 [20], has been limited. Studies have characterised the role of UBO-QIC as a pan G⍺ protein antagonist, demonstrating an inhibitory profile towards Gαq, Gα11 and Gα14 [21], Gα16 [22] and interaction with Gβγ mediated signalling following the activation of the Gαi- coupled receptor [21]. Studies with YM254890 demonstrate that it targets G⍺q/11 however, more recent studies have suggested that it can also target Gαs [23].

GPCR transactivation of the TKR occurs via a completely different mechanism from GPCR transactivation of S/TKR [2,7,8,24,25]. Therefore, we aim to investigate signalling proximal to the receptor by assessing the role of G proteins in GPCR transactivation-dependent signalling. Our pursuit includes identifying a common signalling intermediate that can inhibit all GPCR transactivation-dependent signalling in the context of preventing atherogenic changes on the structure of proteoglycans. With the uncertainty of the specificity of the pharmacological agents, it is apparent that molecular approaches are undoubtedly required to define the role of Gα proteins in transactivation-dependent signalling.

## 2. Results

### 2.1. The Gαq Is Involved in Thrombin-Induced PAR-1 Transactivation of the TGFBR1 and EGFR

Thrombin, via its receptor PAR-1, leads to the transactivation of the TGFBR1 and EGFR [7]. We demonstrate that activation of the two receptor pathways occurs via distinct mechanisms [8]. In the current work, we sought to explore the role of Gαq proteins in PAR-1 transactivation-dependent signalling. We utilised Gαq/11 inhibitor, YM254890 [20], and pan Gαq inhibitor, UBO-QIC [19].

**Figure 1:**
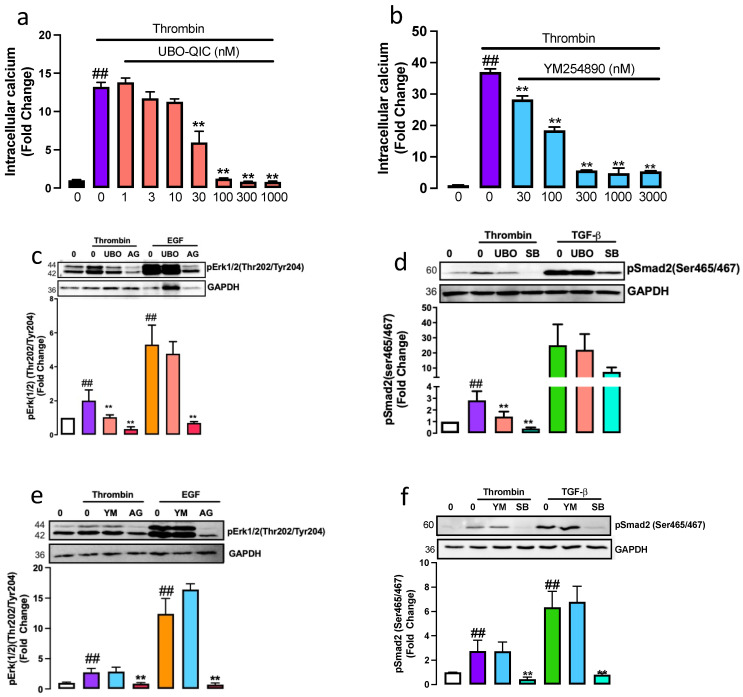
Gαq is involved in PAR-1 mediated transactivation of the TGF-β and EGF receptors. To measure the functionality of respective Gαq inhibitors, thrombin-mediated intracellular calcium release was assessed using FURA-2AM. Cells were preincubation with (**a**) UBO-QIC (UBO) (1–1000 nM) or (**b**) YM254890 (YM) (3–300 nM) followed by Thrombin treatment. Histograms represent fluorescence intensity compared to the basal level. Cells were pre-incubated with (**c**,**e**) UBO-QIC or (**d**,**f**) YM254890 (1 μM) in the presence of thrombin for (**c**,**d**) 15 min or (**d**,**e**) 240 min. (**c**,**d**) EGF for 5 min or (**d**,**e**) TGF-β 10 mins in the presence and absence of respective receptor antagonists AG1478 (AG) or SB431542 (SB). Membranes were incubated with (**c**,**d**) anti-phospho-Erk1/2(Thr202/Tyr204) or (**e**,**f**) anti-phospho-Smad2(Ser465/467). Histograms represent band density expressed as fold per basal. Statistical significance represents ## basal versus agonist *p* < 0.01 and ** agonist versus antagonist *p* < 0.01.

To establish the pharmacological profile of UBO-QIC and YM254890 in human VSMCs, we measured thrombin-mediated intracellular calcium release, a known Gαq response [26]. VSMCs treated with thrombin resulted in a 13-fold (*p* < 0.01) increase in the release of calcium (Figure 1a). In the presence of UBO-QIC (1–1000 nM), we observed a dose-dependent inhibition with 65% inhibition observed at 30 nM and complete inhibition of thrombin-mediated intracellular calcium release at 100–1000 nM (*p* < 0.01). YM254890, dose-dependently inhibited thrombin-mediated intracellular calcium release (Figure 1b) with a 50% inhibition observed at 100 nM and an 85% inhibition observed at 300–3000 μM. This data demonstrated that both UBO-QIC and YM258490 potently inhibit thrombin-mediated intracellular calcium release.

To assess the role of Gαq protein in thrombin transactivation of the EGFR, downstream kinase phospho-Erk1/2 was measured [27]. VSMCs treated with thrombin for 15 min showed a 2-fold (*p* < 0.01) increase of phospho-Erk1/2 (Figure 1c) that was almost completely inhibited in the presence of UBO-QIC (*p* < 0.01). The well-characterised EGFR antagonist, AG1478, used as a control, completely inhibited (*p* < 0.01) thrombin-mediated phospho-Erk1/2. To investigate the role of UBO-QIC on direct EGFR signalling, VSMCs were treated with EGF in the presence and absence of UBO-QIC and AG1478. As expected, EGF stimulated a 5-fold increase in phospho-Erk1/2 (Figure 1c) that was unaffected by UBO-QIC and completely inhibited by AG1478. To assess the role of Gαq/11 in thrombin transactivation of the EGFR, VSMCs were pre-treated with YM254890 (Figure 1d). Thrombin-mediated phospho-Erk1/2 was unaffected in the presence of YM254890; however, as previously reported, thrombin-mediated phospho-Erk1/2 was completely inhibited in the presence of AG1478, and as expected, YM254890 did not affect direct EGFR signalling (Figure 1d). We observed inhibition of thrombin-mediated phospho-Erk with pan Gαq inhibitor, UBO-QIC, but not with YM254890. These data show that the Gαq family is involved in PAR-1 transactivation of the EGFR, leading to phospho-Erk.

A similar approach was utilised to assess the contribution of Gαq on PAR-1 transactivation of the TGFBR1 measured as the phosphorylation of Smad2 in the carboxyl-terminal (phospho-Smad2C). VSMCs treated with thrombin for 240 min showed a 3-fold increase (*p* < 0.01) in phospho-Smad2C (Figure 1e), which was inhibited by 75% to 1.2-fold (*p* < 0.01) in the presence of UBO-QIC. TGFBR1 antagonist, SB431542, used as a control, abolished the thrombin-mediated increase in phospho-Smad2C (*p* < 0.01). To evaluate any possible direct effects of the Gαq inhibitors on TGFBR1 signalling, VSMCs were treated with TGF-β in the presence and absence of UBO-QIC and SB431542. TGF-β treated cells showed a 30-fold increase in the phospho-Smad2C that was unaffected by UBO-QIC (Figure 1e) and inhibited by the TGFBR1 antagonist, SB431542. Thrombin-mediated phospho-Smad2 was unaffected in the presence of YM254890; however, the response was completely inhibited in the presence of SB431542 (Figure 1f). We show that treatment with pan Gαq inhibitor UBO-QIC inhibits thrombin-mediated phospho-Smad2 but is unaffected by YM254890. These results demonstrate that the Gαq family is involved in PAR-1 transactivation of the EGFR and TGFBR1 in VSMCs.

### 2.2. Gαq Is Involved in PAR-1 Mediated mRNA Expression of GAG Enzymes and GAG Chain Elongation

**Figure 2:**
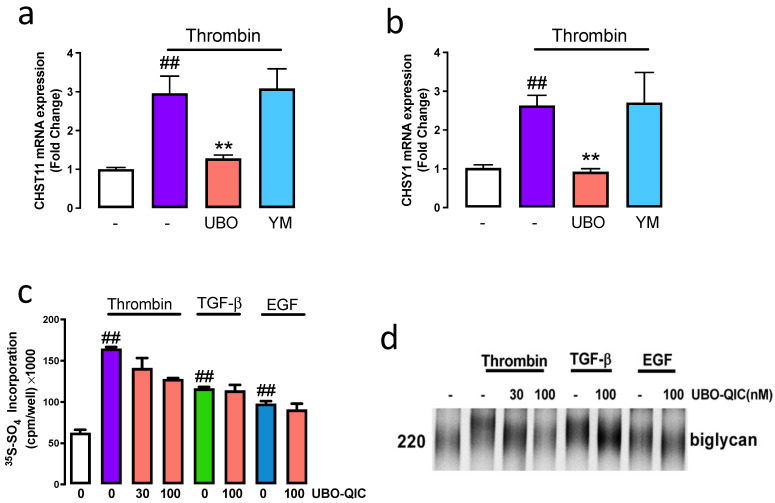
Gαq is involved in PAR-1 regulation of mRNA expression CHST11 and CHSY1, proteoglycan synthesis and GAG chain elongation. VSMCs were pre-incubated with UBO-QIC (UBO), or YM254890 (YM), for 30 min and then exposed to thrombin for 6 h. The mRNA expression of (**a**) CHST11 and (**b**) CHSY1 were assessed using RT-PCR. VSMCs were pre-incubated with UBO-QIC in the presence and absence of thrombin, TGF-β or EGF for 24 h with [^35^S]-sulphate (50 μCi/mL). (**c**) Medium containing secreted proteoglycans were harvested and spotted onto 3 MM paper and were quantitated by CPC precipitation. The histogram shows the fold-change compared to the basal. (**d**) Secreted proteoglycans were isolated using ion exchange chromatography and electrophoresed on a 4–13% SDS-PAGE. Results are the mean and standard error from three separate experiments. Statistical significance analysis is represented with ##, *p* < 0.01 basal versus thrombin and **, *p* < 0.01 thrombin versus antagonists.

Thrombin acting via PAR-1 stimulated the mRNA expression GAG synthesising genes CHST11 and CHSY1 [7,8] and GAG chain modification [6,15]. We utilised UBO-QIC and YM254890 to assess the role of G⍺ proteins in PAR-1 mediated GAG chain modification. VSMCs treated with thrombin resulted in a 3-fold increase in the mRNA expression of CHST11 (Figure 2a). Complete inhibition of thrombin stimulation of CHST11 was observed in the presence of UBO-QIC, however, no change was observed with YM254890 (Figure 2a). A similar response was observed when we studied the mRNA expression of CHSY1. Thrombin-treated cells showed a 2.7-fold (*p* < 0.01) increase in CHSY1 expression (Figure 2b) which was completely inhibited in the presence of UBO-QIC and unaffected in the presence of YM254890 (Figure 2b). This data indicates that PAR-1 mediated GAG gene expression is regulated by the Gαq family.

[^35^S]-Sulphate incorporation can be used to metabolically quantitate the synthesis of GAG chains on proteoglycans. VSMCs treated with thrombin showed a 3-fold (*p* < 0.01) increase of [^35^S]-sulphate incorporation into secreted proteoglycans compared to untreated control (Figure 2c). Thrombin mediated [^35^S]-sulphate incorporation was inhibited by 30% in the presence of UBO-QIC. VSMCs were treated with TGF-β or EGF in the presence and absence of UBO-QIC. As expected, TGF-β and EGF mediated [^35^S]-sulphate incorporation was unaffected in the presence of UBO-QIC (Figure 2c). Isolated proteoglycans were separated by SDS-PAGE the electrophoretic mobility correlates with biglycan size. We show that treatment with thrombin showed a decrease in electrophoretic mobility of biglycan which demonstrates that the biglycan size was larger (Figure 2d). In presence of UBO-QIC the electrophoretic mobility of biglycan increased, indicative of a reduction in biglycan size. TGF-β and EGF showed a decrease in biglycan electrophoretic mobility, unaffected in the presence of UBO-QIC. These results demonstrate that thrombin-mediated GAG chain synthesis and elongation is dependent on the Gαq family.

### 2.3. Thrombin-Mediated GAG Chain Elongation Is Regulated by Gαq but Not Gα11

**Figure 3:**
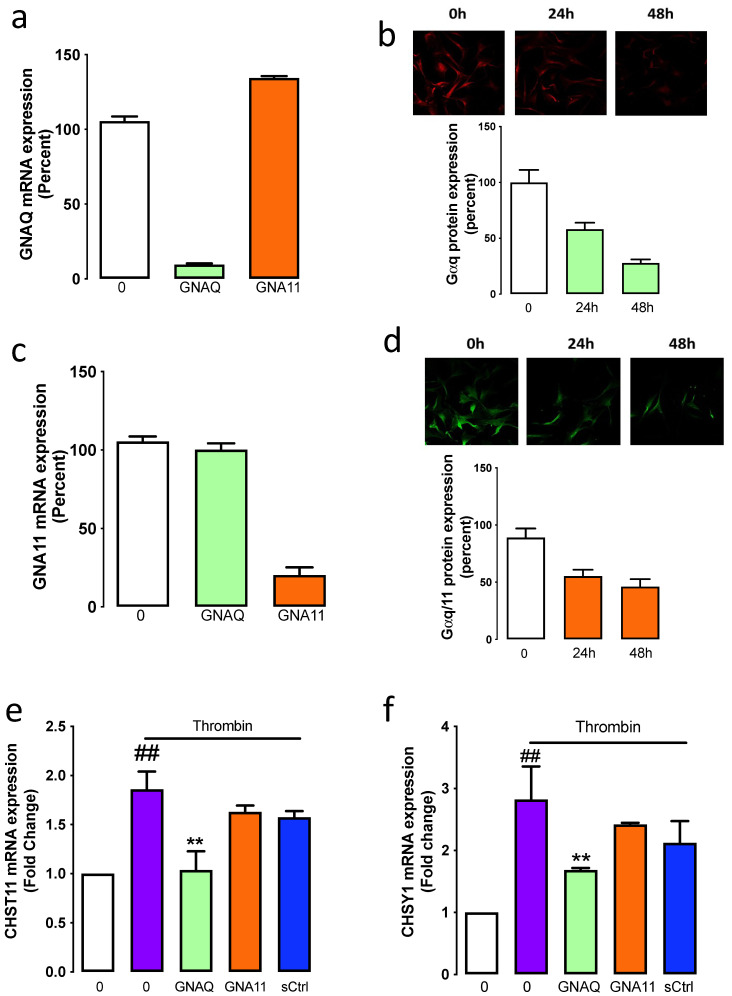
Silencing of GNAQ but not GNA11 is associated with thrombin-mediated stimulation of CHSY1, and CHST11. GNAQ and GNA11 were silenced in VSMCs. The mRNA expression of (**a**) GNAQ and (**c**) GNA11 were analysed after 48 h post-transfection. Data are expressed as the mean and standard error of the mean from two independent experiments. Fixed cells were incubated with (**b**) anti-Gαq or (**d**) anti-Gαq/11 overnight, followed by goat anti-rabbit conjugated with Alexa488 or Alexa594. Images are representative of two independent experiments. Histograms represent the mean intensity of cells presented in 5 field views. Data expressed as mean ± standard error of the mean. The mRNA expression of (**e**) CHST11 and (**f**) CHSY1 were assessed in GNAQ, GNA11 or scramble siRNA (sCtrl) silenced cells treated with Thrombin. Results are the mean and standard error from three separate experiments. Statistical significance was determined by one-way ANOVA, followed by least significant difference post hoc analysis ##, *p* < 0.01 basal versus thrombin and **, *p* < 0.01 thrombin versus antagonists.

We have thus far utilised a pharmacological approach to demonstrate that thrombin-mediated GAG chain elongation is dependent on the Gαq family. To further explore this finding, we have used a molecular approach to investigate the contribution of Gαq (GNAQ) and Gα11 (GNA11) on thrombin signalling and the stimulation of GAG synthesising enzyme expression. The silencing of GNAQ with siRNA resulted in a 90% reduction of GNAQ mRNA expression (Figure 3a), and by silencing GNA11, we observed an 80% knockdown in GNA11 mRNA expression (Figure 3b). The silencing of GNA11 had no effect on GNAQ expression, and the silencing of GNAQ had no impact on GNA11 expression, which demonstrates that although the two isoforms share 88% homology at the protein level, there was no crossover in the knockdown targets of the siRNA.

To demonstrate that silencing of the mRNA expression resulted in knockdown at the protein level, we measured G⍺q and G⍺11 protein expression 24- and 48 h post-transfection using immunofluorescence. Silencing of GNAQ knocked down G⍺q protein expression by 50% and 80% at 24- and 48 h post-transfection, respectively (Figure 3c), and siRNA specific to GNA11 led to a 50% knockdown of Gα11 protein expression 24 h post-transfection, and 65% knockdown was observed at 48 h (Figure 3d). These data demonstrate that silencing of the mRNA expression of GNAQ and GNA11 resulted in a decreased expression at the protein level.

To assess the functional consequences of the specific Gα protein knockdown, VSMCs were treated in the presence and absence of thrombin and siRNA to GNAQ or GNA11 and the mRNA expression of CHSY1 and CHST11 was measured. Thrombin treatment stimulated a 2.8-fold increase (*p* < 0.01) in the mRNA expression of CHSY1 (Figure 3e), which was inhibited by 50% to 1.6-fold (*p* < 0.01) in the GNAQ silenced cells; however, silencing of GNA11 had no effect. A similar pattern was observed with thrombin-mediated mRNA expression of CHST11. Thrombin-stimulated CHST11 mRNA expression was inhibited entirely in GNAQ silenced cells; however, CHST11 mRNA expression was unaffected in GNA11 silenced cells (Figure 3f). These data demonstrate that thrombin-mediated mRNA expression of CHST11 and CHSY1 occurs via G⍺q but not G⍺11-dependent pathways.

## 3. Discussion

GPCR transactivation-dependent signalling is an important contributor to GPCR signalling and accounts for as much as 50% of signalling outputs [9]. Focusing on transactivation-dependent signalling, we [8,15,28] and others [29,30] have shown that the biochemical mechanisms of GPCR transactivation of kinase receptors are distinct. One element of GPCR transactivation of the EGFR occurs via the triple membrane bypass mechanism, whereas transactivation of the TGFBR1 via ROCK/Integrin-dependent pathways [31]. We have reported that in human VSMCs, thrombin via PAR-1 transactivates the EGFR and TGFBR1, leading to an increase in the expression of the rate-limiting enzymes associated with GAG chain elongation [7] and proteoglycan synthesis [15]. Modified GAG chains on proteoglycans are related to the retention of lipids in the vessel wall of large blood vessels as one of the earliest steps in atherogenesis [32]. Hence, finding a common target could account to block all thrombin-mediated GAG chain modification and would represent a potential therapeutic target to prevent early lipid deposition within the vessel wall [32]. We have demonstrated that almost identical G proteins can have specific roles in cell biology. There are examples of drugs that can target some of the G proteins, so our results indicated that pharmacological and pharmaceutical development which produces specific inhibition of individual G protein isoforms might lead to the discovery and introduction of whole new classes of drugs to expand the already enormous role of GPCRs in human therapeutics.

Our data using pharmacological and molecular tools demonstrates that PAR-1 transactivation of the EGFR and TGFBR1 leading to GAG chain elongation occurs specifically via G⍺q but not G⍺11-dependent pathways. These studies identify the Gαq as a central integrating point for all PAR-1 transactivation-dependent signalling leading to proteoglycan synthesis and GAG chain elongation and a highly specific target for the prevention of atherosclerosis. This would be particularly pertinent if the observation for thrombin/PAR-1 could be extrapolated to other GPCRs such as endothelin [33,34] and LPA [28,35] which are associated with vascular pathology and GAG chain elongation.

The limited availability of specific antagonists of G proteins has hindered the study of G proteins in transactivation-dependent signalling. We have reviewed the tools used to evaluate the role of G proteins in GPCR signalling in detail (see [17,18]). UBO-QIC and YM254890 have had variable and limited availability over the last few decades and have only recently (last five years) become commercially available. YM254890 and UBO-QIC share a similar chemical structure [23,36]. Comprehensive characterisation studies have revealed that UBO-QIC (FR900359) is a pan Gαq inhibitor that is not selective to the isoforms [37,38]. Use of pan Gαq member inhibitor UBO-QIC, we show that the Gαq protein regulated PAR-1 transactivation of kinase receptors and GAG chain elongation. Although YM25480 inhibits thrombin-mediated intracellular calcium release, it had no effect on transactivation-dependent signalling and GAG chain elongation. Complementary molecular studies took a deeper look at the most abundantly expressed Gαq members (Gαq and Gα11). Although the two Gαq members share 90% homology, we did not observe any crossover knockdown on mRNA expression of GNAQ or GNA11. We did observe that the two Gαq members had different signalling profiles where only the Gαq but not the Gα11 was involved in regulating PAR-1 mediated GAG chain elongation.

We show that Gαq member is the central integrating point for GPCR transactivation-dependent signalling. In keratinocytes [39], rat myoblasts [40], and CHO cells [41], GPCR transactivation of TKRs was completely inhibited in the presence of YM254890. In contrast, more recent studies with Angiotensin II [42] have revealed that Angiotensin II receptor transactivation of the EGFR is independent of G⍺q/11 pathways. In our VSMC model, we show that thrombin-mediated transactivation of the EGFR was unaffected in the presence of YM254890, however, attenuated by pan Gαq inhibitor UBO-QIC, demonstrating no role for Gα11 in PAR-1 transactivation of EGFR. Consistent with our observations, LPA-mediated transactivation of the TGFBR1 was attenuated by Gαq following pharmacological and molecular silencing of mouse embryonic cells [43] and epithelial cells [44,45]. We show that in human VSMCs, PAR-1 mediated transactivation of the TGFBR1 and EGFR leads to GAG chain elongation occurring specifically via Gαq but not Gα11 dependent pathways. Our findings highlight that the Gαq is the common central mechanism for PAR-1 transactivation-dependent signalling in VSMCs.

Several pharmacological and molecular approaches have been used to assess G⍺q in the vasculature. Targeting the G⍺q in overt heart failure improved contractile dysfunction in the myocardium and improved/reversed heart failure [46]. Pharmacological interventions using YM254890 reduced shear stress-induced thrombus formation in the cynomolgus monkey [47] and electrically induced arterial thrombosis in a rat model [20]. Following vascular injury, YM254890 inhibited neointima formation; however, it prompted hypotensive effects in vivo [48]. We show that targeting the G⍺q but not the G⍺11 reduced the expression of the rate-limiting enzymes associated with elongating GAG chains on lipid binding proteoglycans. These data would be particularly pertinent if the observation for thrombin/PAR-1 could be extrapolated to the many GPCRs associated with vascular pathologies such as angiotensin II, endothelin-1 and LPA.

Gαq and Gα11 are 90% similar in their amino acid sequence. We elegantly show that the two isoforms exhibit different functional roles. We use a pan Gαq inhibitor to identify a role for G proteins in GPCR transactivation-dependent signalling and a molecular approach to differentiate between the specific Gαq members. Our work highlights the Gαq member as a common signalling intermediate for GPCR transactivation of kinase receptors. GPCRs are targets for approximately 30% of the global therapeutic market, and we show that targeting specific isoforms can regulate different cellular responses, therefore, targeting specific G proteins could reduce off-target effects. Our findings highlight a need for pharmacological development to produce tools for specific inhibition of individual G proteins to allow for deeper insights into the enormous role of GPCR in human pathophysiology.

## 4. Materials and Methods

### 4.1. Materials

Dulbecco’s Modified Eagle medium (DMEM), antibiotics (10,000 U/mL penicillin, 10,000 μg/mL streptomycin), foetal bovine serum (FBS), Alexa Fluor 488 and 594 goat anti-mouse antibodies, FURA-2 AM ester were purchased from Thermo Fisher Scientific (Scoresby, VIC, Australia). SB431542, AG1478, and EGF were purchased from Sigma-Aldrich (Castle Hill, NSW, Australia). Primers for *18S*, *GNAQ*, *GNA11*, *CHSY1* and *CHST11*, RNeasy Mini Kit, QuantiTect Reverse Transcription Kit, and QuantiNova SYBR green RT-PCR kit were purchased from Qiagen (Clayton, VIC, Australia). Human transforming growth factor beta-1, antibodies to phospho-Smad2(Ser465/467), phospho-Erk1/2(Thr202/Tyr204), and glyceraldehyde-3-phosphate dehydrogenase (GAPDH) (HRP conjugate) were purchased from Australian Bioresearch (Balcatta, WA, Australia). SCH79797 was from In Vitro Technologies (Noble Park, VIC, Australia). YM254890 was obtained from Focus Bioscience (Murarrie, QLD, Australia). Polyvinylidene fluoride (PVDF) Tween 20, 30% acrylamide/Bis Solution and Image Lab imaging software were from BioRad Laboratories (South Granville, NSW, Australia). Carrier-free [^35^S]-SO4 was purchased from PerkinElmer (Glen Waverley, VIC, Australia). Cetylpyridinium chloride was purchased from Unilab Chemicals & Pharmaceuticals Pvt. Ltd. (Mumbai, India). Human coronary artery smooth muscle cells, AD1 4D-Nucleofector™ Y kit, smooth muscle growth medium-2 bullet kit and SingleQuot kit supplements and growth factors were from LONZA (Mount Waverley, VIC, Australia). SiGENOME Human GNA11 siRNA (SMARTpool), SiGENOME Human GNAQ siRNA (SMARTpool) and siGENOME non-targeting siRNA (SMARTpool) were purchased from Millennium Science (Mulgrave, VIC, Australia). Antibodies to GNAQ (ab75825) and GNA11 (ab192507) were purchased from Abcam (Melbourne, VIC, Australia).

### 4.2. Cell Culture

VSMCs were isolated using the explant technique from discarded segments of the saphenous veins from patient donors undergoing surgery at Alfred Hospital (Melbourne, VIC, Australia) [49]. Cells were grown in DMEM (10% FBS and 1% antibiotics) at 37 °C in 5% CO_2_. VSMCs were seeded in 96-, 24-, 12- and 6-well plates. Cells were grown to confluence and then rendered quiescent by serum deprivation for either 24 or 48 h. Human coronary artery smooth muscle cells (CASMCs) were purchased from Lonza and grown according to the instructions from the manufacturer. The treatment conditions are detailed in the respective figure legends.

### 4.3. Quantification of Intracellular Calcium Release

VSMCs grown in 96-well plates were loaded with FURA-2AM ester (2 µM for 30 min). Dye loading and experiments were performed in Hanks buffer (10 mM HEPES, 140 mM NaCl, 5 mM KCl, 2 mM CaCl2, 1 mM MgCl2, 11 mM glucose). The cells were pre-incubated with antagonists for 30 min before agonist injection. Fluorescence was measured at 340 nm and 380 nm excitation and 510 nm emission wavelengths using a Flexstation 3 plate reader (Molecular Devices). The sustained calcium response was calculated as the difference between basal and peak fluorescence after agonist stimulation.

### 4.4. Western Blot Analysis

Whole-cell lysates (40 µg) were separated on 10% SDS-PAGE and transferred onto PVDF membranes. Membranes were blocked with 5% BSA, then incubated with a primary antibody targeting the protein of interest, followed by HRP-anti-rabbit IgG and ECL detection. Blots were imaged using the Bio-Rad gel documentation system (ChemiDoc MP), and densitometry analysis was performed with Image Lab (Bio-Rad) 5.2.1.

### 4.5. Quantitative Real-Time-Polymerase Chain Reaction Analysis

Total RNA was isolated from cells with the RNeasy Mini kit according to the instructions of the manufacturer. RNA concentration and purity were assessed by Nanodrop2000 spectrophotometer. cDNA (1000 ng) was synthesised using a Quantitect reverse transcriptase kit. Quantitative RT-PCR was performed using Qiagen Rotor-Gene Q and QuantiNova SYBR green PCR master mix kit. Data were normalised to the ribosomal 18S housekeeping gene to adjust for controlling variations between individual experiments. Relative expression of mRNA levels was quantified using the comparative delta-delta Ct method.

### 4.6. Quantification of Radiolabel Incorporation into Proteoglycans

Quiescent cells were labelled with 50 μCi/mL [^35^S]-sulphate in the presence or absence of agonists or antagonists for 24 h. Secreted proteoglycans were harvested from treated cells in the presence of 5 mM benzamidine in 0.1 M 6-aminocaproic acid to prevent degradation. Incorporation of the radiolabel into proteoglycans was measured by CPC precipitation assay as previously described [50]. Labelled proteoglycans were isolated by DEAE-Sephacel anionic exchange mini-columns [50,51]. Proteoglycans labelled with [^35^S]-sulphate were separated on 4–13% acrylamide gels. The gels were processed and then developed on a Cylone Plus Phosphor Imager (Perkin Elmer).

### 4.7. Nucleofection Using siRNA

The Lonza AD1 4D-Nucleofectory Y kit was used according to the instruction of the manufacturer. siRNA to GNAQ and GNA11 at 105 pmol was utilised. The 24-well plate containing the siRNA, supplement and dipping electrode array was placed in 4D-Nucelofector core Y-unit using ER137 pulse. The siRNA substrate solution was replaced with a fresh medium, and cells were incubated for 36 h (unless stated otherwise) before additional treatment.

### 4.8. Immunofluorescent Imaging

Cells were grown and treated on glass coverslips and then fixed with 2% paraformaldehyde in 1 × PBS. The coverslips were washed with 1 × PBS and blocked with 10% horse serum for 30 min. Cells were incubated with primary antibody overnight in a humified chamber followed by fluorophore-conjugated secondary for 1 h. Coverslips were incubated with Hoechst stain for 30 min, then mounted on slides and dried overnight at 4 °C. Cells were imaged using a Nikon C1 confocal microscope.

### 4.9. Statistical Analysis

Normalised data are expressed as the mean ± standard error of the mean of four independent experiments unless stated otherwise. A one-way ANOVA was used to calculate the statistical significance of normalised data, followed by the least significant difference posthoc analysis. Results were considered significant when the probability was less than 0.05 (* *p* < 0.05) and 0.01 (** *p* < 0.01).

## Data Availability

Data can be made available upon request.
